# Exploring the Potential of Phytocompounds for Targeting Epigenetic Mechanisms in Rheumatoid Arthritis: An In Silico Study Using Similarity Indexing

**DOI:** 10.3390/molecules28062430

**Published:** 2023-03-07

**Authors:** Sanjay H. Deshpande, Zabin K. Bagewadi, T. M. Yunus Khan, Mater H. Mahnashi, Ibrahim Ahmed Shaikh, Sultan Alshehery, Aejaz A. Khan, Vishal S. Patil, Subarna Roy

**Affiliations:** 1 Department of Biotechnology, KLE Technological University, Hubballi 580031, India; 2 Department of Mechanical Engineering, College of Engineering, King Khalid University, Abha 61421, Saudi Arabia; 3 Department of Pharmaceutical Chemistry, College of Pharmacy, Najran University, Najran 66462, Saudi Arabia; 4 Department of Pharmacology, College of Pharmacy, Najran University, Najran 66462, Saudi Arabia; 5 Department of General Science, Ibn Sina National College for Medical Studies, Jeddah 22421, Saudi Arabia; 6 ICMR—National Institute of Traditional Medicine, Belagavi 590010, India

**Keywords:** similarity indexing, rheumatoid arthritis, molecular docking, molecular dynamics, traditional medicine, *Aglaia leptantha*, *Aglaia edulis*

## Abstract

Finding structurally similar compounds in compound databases is highly efficient and is widely used in present-day drug discovery methodology. The most-trusted and -followed similarity indexing method is Tanimoto similarity indexing. Epigenetic proteins like histone deacetylases (HDACs) inhibitors are traditionally used to target cancer, but have only been investigated very recently for their possible effectiveness against rheumatoid arthritis (RA). The synthetic drugs that have been identified and used for the inhibition of HDACs include SAHA, which is being used to inhibit the activity of HDACs of different classes. SAHA was chosen as a compound of high importance as it is reported to inhibit the activity of many HDAC types. Similarity searching using the UNPD database as a reference identified aglaithioduline from the *Aglaia leptantha* compound as having a ~70% similarity of molecular fingerprints with SAHA, based on the Tanimoto indexing method using ChemmineR. Aglaithioduline is abundantly present in the shell and fruits of *A. leptantha*. In silico studies with aglaithioduline were carried out against the HDAC8 protein target and showed a binding affinity of −8.5 kcal mol. The complex was further subjected to molecular dynamics simulation using Gromacs. The RMSD, RMSF, compactness and SASA plots of the target with aglaithioduline, in comparison with the co-crystallized ligand (SAHA) system, showed a very stable configuration. The results of the study are supportive of the usage of *A. leptantha* and *A. edulis* in Indian traditional medicine for the treatment of pain-related ailments similar to RA. Our study therefore calls for further investigation of *A. leptantha* and *A. edulis* for their potential use against RA by targeting epigenetic changes, using in vivo and in vitro studies.

## 1. Introduction

Rheumatoid arthritis (RA) is an autoimmune disease characterized as a chronic inflammatory disease, which affects around about 1% of the population. It is mainly caused by genetic dispositions and environmental conditions, but may also occur because of abnormal activation of the immune system. The divergent activation of innate as well as adapted immune systems plays an important role in the pathogenesis of RA. Elevated levels of inflammatory cytokines produced from B cells and T cells play a very significant role in the development of the disease. The increase in levels of cytokines creates an abnormal environment around the cartilage and bone cells, leading to their destruction, which creates a disturbance in the moment of peripheral joints. The main instances that define RA in its active state are swelling and joint pain, resulting in disability and the destruction of joints, which finally leads to dysfunction of the joints. The anomalies that are characteristic of RA include the erosion of joints immediately after the symptoms appear, synovial infiltration in the clinically unaffected joints and the presentation of autoantibodies before the beginning of the disease, suggesting that the development of the disease occurs significantly earlier than the clinically significant symptoms start to appear [1].

Technological developments have led to clearer identification of the pathogenesis of RA. In recent years, the contribution of resident synovial fibroblasts (SF) has emerged as a key component in the pathogenesis and development of RA leading to the destruction of joints. RASF (rheumatoid arthritis synovial fibroblasts) are the most common cell types at the site of the invasion [2]. In recent years, aberrant epigenetic changes are characterized in connection with RASF that can help in solving intrinsic activation during the destruction of joints. This connection can help in providing the missing link between RA, risk factors and different therapy response [3]. Epigenetic modifications can be defined as the alteration of gene expression or phenotypes at the cellular level caused by mechanisms other than those of changes occurring in the DNA sequence where these modifications can be induced by environmental changes that are short-lived and reversible alterations [4]. Epigenetic modifications include DNA methylation and a network of post-translational modifications on histone tails like acetylation, phosphorylation, methylation, ubiquitination or sumoylation [5].

The target HDAC8 (histone deacetylase 8) is involved in the reaction that catalyzes the deacetylation of lysine residues present on the N-terminal region of the core histones [6,7,8]. The deacetylation functionality of the HDAC (histone deacetylase) enzymes plays a key role in epigenetic repression, which directly affects the transcriptional regulation and cell cycle development [6,7,9]. The key structural configuration of HDAC8 consists of a specific domain (region) ranging from 14–324 amino acids, which defines it as histone deacetylase enzyme [10], with an active site in 143rd position, and two types of binding sites: a divalent metal cation binding site in 178th, 180th and 267th position [11]; and substrate binding site in 101st, 151st and 306th position, as shown in Figure S1. Furthermore, disease-modifying antirheumatic drugs (DMARDs) are used specifically for the treatment of RA. Drugs like Methotrexate, Hydroxychloroquine, Sulfasalazine and gold salts are the most commonly used DMARDs, but due to their high number of side effects, which include damage to bone marrow and the nervous system, there is a need for alternative therapeutic procedures for the treatment of RA [12]. The mechanism of epigenetic modification in RA has gained growing research interest in recent times. The application of epigenetically modified methods is an important field in the research of RA pathogenesis. SAHA (Suberanilohydroxamic acid) is one of the known inhibitors of HDAC that has been applied in the treatment of RA and is known to be effective [13]. In this study, the compound aglaithioduline was selected based on the similarity indexing approach following most of the parameters considered.

The information on plants that are being used for the treatment of rheumatoid arthritis was obtained from multiple sources like Indian traditional knowledge, Ayurveda practitioners and some published material available globally. Table S1 provides a list of published plants given in sources like “Indian Medicinal Plants, An Illustrated Dictionary”, C.P. Khare, 2007, Springer-Verlag New York [14] and “WHO Monographs on Selected Medicinal Plants”—Volume 1, 2, 3, 4.

Chemistry is a field of study where structural analogy plays a very important role, and understanding the analogy and its functional impact becomes very important. Medicinal chemistry would have been very difficult to study, understand and apply if the structural similarity principle did not exist. The similar property principle states that structurally similar molecules tend to have similar properties, making this method a rule of thumb for application where there is an absence of detailed knowledge of chemicals. Similarity indexing mainly focuses on chemical similarity, which has also increased interest in the field of biological similarity. Similarity measurement techniques have always been looked at as foreign techniques, and people still are apprehensive about their efficiency and credibility. A single measure cannot therefore be stated as a perfect measure of similarity [15]. Molecular descriptors are the numerical values that have been assigned to a chemical structure, and the level of dimensional properties is defined by these descriptors, as shown in Table S2.

The similarity coefficient is a quantitative measure of similarity between two sets of molecular descriptors. The similarity coefficient can be measured by various methods like the Tanimoto coefficient/fingerprint method, the cosine coefficient method, the Euclidean distance method and the Tversky index. The Tanimoto fingerprint method is the standard method for measuring the similarity coefficient, which is accepted globally, as given in Formula (1).

The Tanimoto coefficient for two molecules, *A* and *B*, can be given as:

(1) $$SIM_{AB}=\frac{c}{a+b-c}$$

where the *c* bits are set in common in the two fingerprints, and the *a* and *b* bits are set in the fingerprints for *A* and *B* respectively [16,17].

The need to validate the application of traditionally used herbs in a medicinal system with modern techniques is a very important step for the acceptance of these herbs globally. The gap between the usage and validation of traditional medicines can be closed only by initiating preliminary studies. In the current study, one such method of similarity indexing, in combination with widely used in silico techniques like molecular docking and molecular dynamics (MD), has been carried out [18,19]. Along with MD, MM/PBSA analysis and principal component analysis were carried out to understand the ligand–protein complex mechanisms in a system [20]. The main objective of this study is to understand the similarity correlation between the compounds from traditionally used herbs and standard drugs in the treatment of rheumatoid arthritis.

## 2. Results

### 2.1. Similarity Indexing

Similarity searching using the R programming method and the Tanimoto coefficient method resulted in similarity indices of phytocompounds in comparison to SAHA based on fingerprint values. The similarity indexing was carried out using the Shiny application called “Similarity indexing”, hosted and available for public usage on GitHub (https://github.com/sandes89/Similarityindexing, accessed on 30 September 2022). The application was used to identify highly similar compounds. Aglaithioduline showed ~70% of similarity in comparison to SAHA, the standard drug, and the co-crystallized compound with HDAC8 (PDB id: 1T69). The molecular properties of the compounds SAHA and aglaithioduline were compared, and it was seen that both compounds showed very similar chemical properties, as shown in Table 1. The pharmacokinetics properties of both compounds were also compared, and it was seen that both the compounds had very similar activities and properties predicted.

Both the compounds, i.e., the standard drug (SAHA) and the compound obtained from similarity indexing (aglaithioduline), were subjected to preADMET checks using a pKcsm server [21], and complete details are given in Supplementary Table S1. From the preADMET studies, it was observed that the water solubility of both compounds showed very similar values, whereas the caco2 permeability of aglaithioduline was higher in comparison with that of SAHA. The total clearance rate, which includes both hepatic and renal clearance, was very high for aglaithioduline, making it more effective in terms of excretion from the body.

### 2.2. Binding Site Assignment

The binding site for docking studies was assigned using the binding sites of the co-crystallized structure, P2RANK and a review of the literature. It was also based on curated sites from UniProt KB (Q9BY41). The assigned sites and the site coordinates are given in Table 2.

### 2.3. Protein–Ligand Interactions

**Figure 1:**
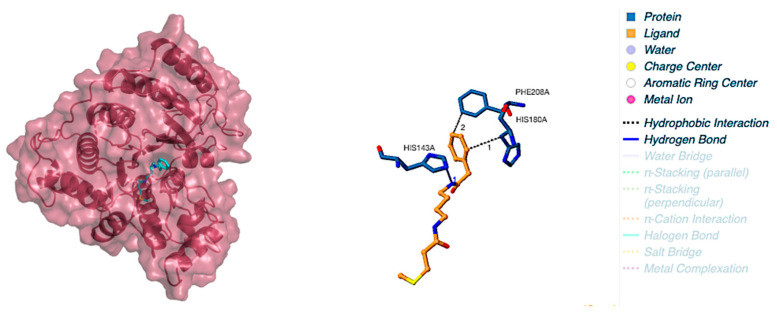
Aglaithioduline in complex HDAC8 target protein with 2D interaction sites.

**Figure 2:**
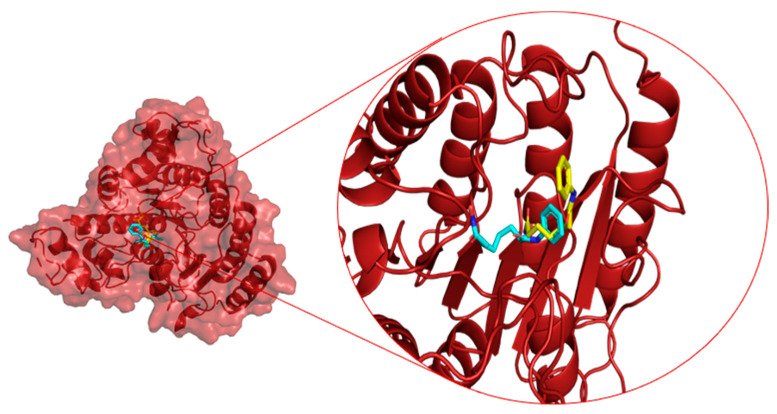
Superimposed structure of HDAC8 with co-crystallized SAHA and docked SAHA. (Cyan: docked pose, Yellow: co-crystallized ligand).

The molecular docking with the shortlisted compound based on the similarity indexing calculation and HDAC8 showed very promising results. The binding score and the interaction of compounds with the amino acid sites of the protein are tabulated in Table 3. Aglaithioduline showed a binding affinity with −8.4 kcal/mol and the interaction diagram is shown in Figure 1. Moreover, to confirm the correctness of the docking, the target HDAC8 and SAHA from the crystallized structure (PDB id: 1T69) were docked using the same configuration. The docking result clearly indicated that the compound stayed in the same binding pocket, thereby confirming the appropriateness and correctness of the docking procedure. The docked and the crystallized structure were superimposed and is shown in Figure 2. The results of docking showed that the binding affinity value of SAHA (standard inhibitor) with the target was found to be highest, and the aglaithioduline binding affinity value was very near to that of SAHA. Aglaithioduline, having ~70% structural similarity with SAHA, showed very promising results. Based on the binding affinity and the interactions, the HDAC8–aglaithioduline complex was further taken for molecular dynamics and simulation studies.

### 2.4. Molecular Dynamics and Simulation

The molecular dynamics trajectory analysis for all three systems, namely APO (protein only), LIG (protein in complex with standard drug SAHA) and AG (protein in complex with aglaithioduline), was carried out. The RMSD of the backbone, RMSF of the residues, solvent-accessible surface area (SASA) and radius of gyration were plotted, and the number of hydrogen bonds between the compound and protein was also plotted for 100 ns (100,000 ps) of simulation duration.

### 2.5. Trajectory Analysis of APO, LIG and AG Systems

**Figure 3:**
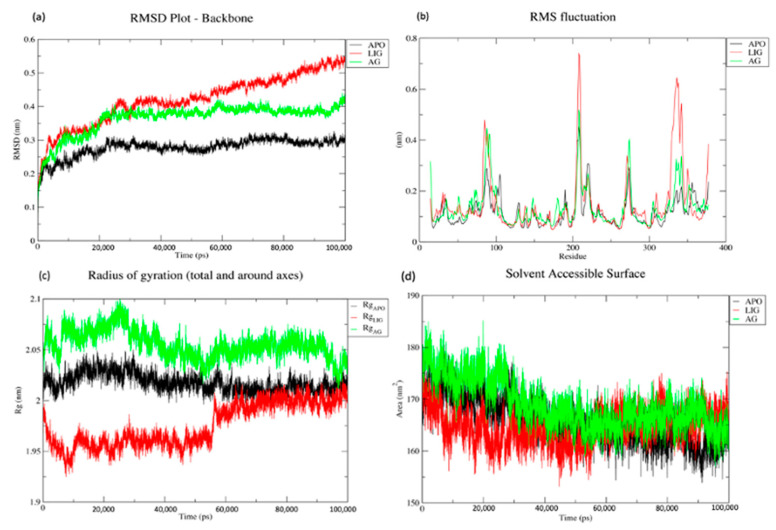
(**a**) RMSD plot for the backbone of APO, LIG and AG systems plotted over 100 ns; (**b**) RMSF plot for protein residues APO, LIG and AG systems over 100 ns; (**c**) compactness measure (radius of gyration) of APO, LIG and AG systems plotted over 100 ns; and (**d**) solvent-accessible surface area (SASA) in nm^2^ for APO, LIG and AG systems over 100 ns. (100 ns = 100,000 ps).

RMSD analysis of the trajectories was carried out and it was observed that the APO system was stable after 20 ns. The RMSD plot of the LIG system and AG system was plotted and it was seen that the RMSD of aglaithioduline was higher than the APO system but stayed stable after the 20ns, and the RMSD was seen to be in the range of 0.3–0.38 nm. The trajectory of the AG system followed the pattern of the APO system (0.2–0.28 nm), whereas the trajectory of the standard drug (LIG system) showed high variability, which showed an increasing trend Figure 3a.

The RMSF of APO, AG and LIG was plotted and it was clearly seen that the APO and AG system showed lower fluctuations than the LIG system. The stable residues indicate the stable configuration of the system in the APO and LIG systems, as shown in Figure 3b.

The Rg, which is a measure of the overall size of a protein, is calculated as the root-mean-square distance of a group of atoms from their shared center of mass. The Rg plot of APO, AG and LIG in Figure 3c displays significant variation and fluctuation during simulation time, which suggests that the native conformation of the protein is flexible and subject to change throughout the simulation period. Therefore, this analysis provides valuable insights into the dimensions and dynamics of the protein structure.

In addition, the solvent-accessible surface of the target protein was calculated and the volume against the time was plotted (Figure 3d). It was seen that the volume of the APO and AG systems stayed similar but the volume of the LIG system varied during the simulation duration.

### 2.6. Hydrogen Bond Counts for LIG and AG Systems

**Figure 4:**
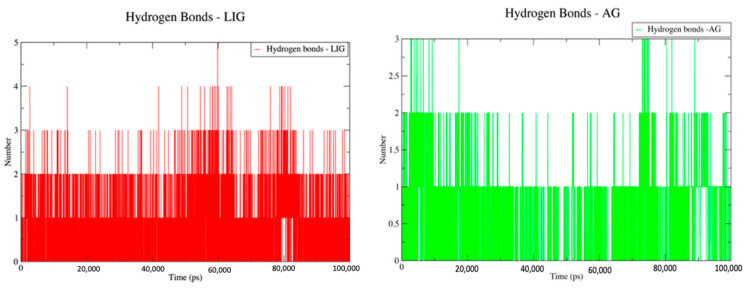
Hydrogen bond contacts for LIG and AG system for the duration of 100 ns (100,000 ps).

Hydrogen bond analysis of the LIG and AG systems was carried out using the “hbonds” module of Gromacs. The analysis clearly showed that the LIG and AG systems had hydrogen bonds in the system throughout the simulation duration. The presence of hydrogen bonds during simulation indicates the stability of the protein in the system, which is a very important aspect in understanding the protein–ligand interactions (Figure 4).

### 2.7. MM/PBSA and Residual Decomposition Energy

**Figure 5:**
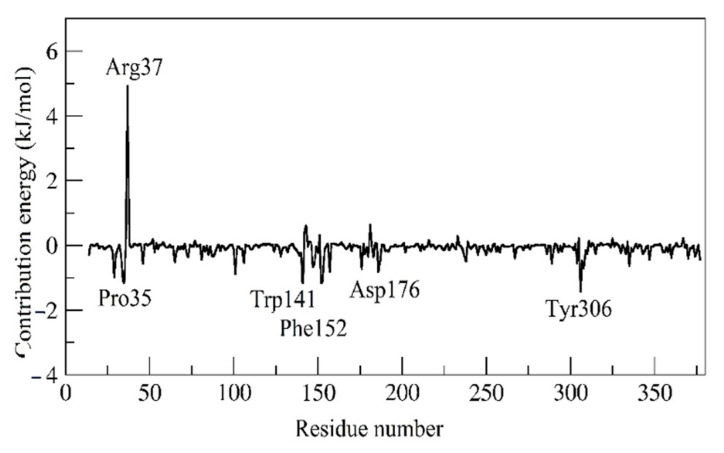
Contribution energy plot highlighting the importance of the AG system binding pocket residues in stable complex formation.

The estimated relative binding energy of the complex AG was −53.405 ± 44.255 kJ/mol and the Van der Waals, electrostatic, polar solvation and SASA energy was −45.480 ± 73.158, −5.641 ± 9.017, 2.539 ± 61.749, and −4.823 ± 7.161 kJ/mol, respectively. Furthermore, the AG system residue decomposition energy was calculated to infer the individual residue contributing most to the binding energy (Figure 5). The residues Pro35, Trp141, Phe152, Asp176 and Tyr306 favored stable complex formation by exhibiting the lowest contribution energy of −1.319, −1.552, −1.449, −0.727, and −1.44kJ/mol, respectively. However, the Arg37 residue did not favor the interactions (5.032 kJ/mol).

**Figure 6:**
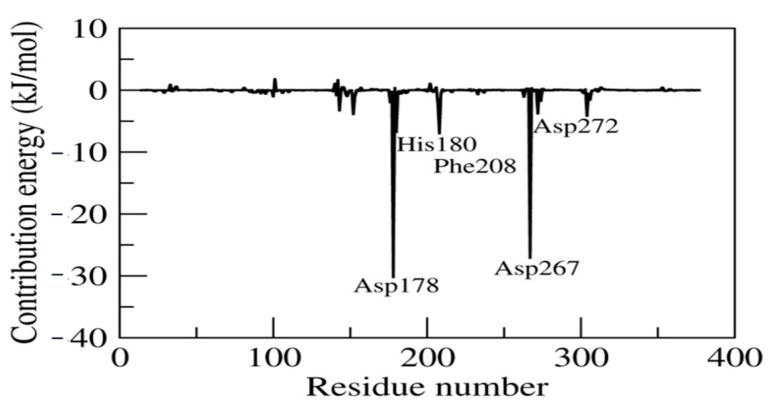
Contribution energy plot highlighting the importance of the LIG system binding pocket residues in stable complex formation.

The estimated relative binding energy of the complex LIG was 4.996 ± 16.014 kJ/mol and the Van der Waals, electrostatic, polar solvation and SASA energy was −112.149 ± 16.106, −96.119 ± 12.946, 226.486 ± 17.539, and −13.222 ± 0.869 kJ/mol, respectively. Furthermore, the LIG system residue decomposition energy was calculated to infer the individual residue contributing most to the binding energy (Figure 6). The residues Asp267, Asp272, Asp178, His180 and Phe208 favored stable complex formation by exhibiting the lowest contribution energy of −27.08, −3.76, −30.19, −6.77 and −6.97 kJ/mol, respectively.

### 2.8. Principal Component Analysis

**Figure 7:**
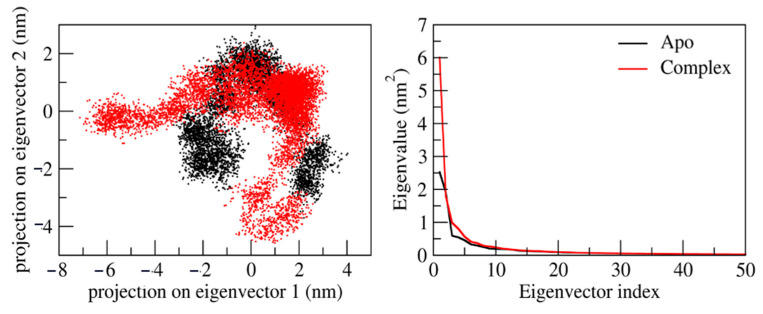
Principal component analysis of protein–ligand complexes: the collective motion of Apo form (black) and ligand-bound (red) using projections of MD trajectories on two eigenvectors corresponding to the first two principal components. The first 50 eigenvectors were plotted versus eigenvalue for Apo (black) and ligand-bound molecule (red).

We performed principal component analysis to explore the conformational flexibility and diversity of conformations that emerged from the stable trajectory obtained from 100ns MD simulation. The maximum collective motion is captured by the first 50 eigenvectors/principal components. Therefore, we precisely studied the first two eigenvectors/PCs (principal components) in detail. Figure 7 represents the 2D projection of the first two eigenvectors. It is observed that the Apo form shows a lower diversity of conformation during the simulations (−3 to 4). However, the ligand–protein complex shows higher diversity of conformations during simulation (−7 to 3). This reveals that the ligand with protein is well equilibrated and stabilized during the simulation.

## 3. Discussion

Many types of similarity indexing methods that utilize 2D fingerprints are available, and each has its own advantages and disadvantages. In this study, the Tanimoto coefficient (TC) is used to quantify and compare the similarity of the drug. The output from the TC is in the range between 0 (maximum dissimilarity) and 1 (maximum similarity) [22]. The TC is considered to be one of the best indices in similarity indexing, and is most efficient in cases that have compounds with moderate molecular weight [22]. The similarity indexing of SAHA and natural compounds resulted in aglaithioduline with a coefficient of ~0.7 (70% similarity).

Aglaithioduline has a molecular weight of less than 300 which is the best-fit compound for the Tanimoto indexing method. The molecular docking studies result in the best-fit pose of aglaithioduline in complex with HDAC8 with a binding energy of −8.5 kcal/mol. The best-fit complex further underwent molecular dynamics analysis. The RMSD plot clearly showed that the LIG system of SAHA was unstable in comparison with the aglaithioduline (AG) system. RMSD stability indicates the stability of the protein–ligand binding and the RMSF calculations also showed stability in the AG system [23]. The MM/PBSA and residual decomposition energy analysis indicated stability and showed low energy, which is highly favorable in nature for the protein–ligand complex. Aglaithioduline, which is present in *Aglaia leptantha* and *Aglaia edulis*, has been traditionally used in Indian traditional medicine for the treatment of cancer, inflammatory conditions, fungal infections, tuberculosis and viruses [24,25]. Moreover, specific plants from *Aglaia* have been known to inhibit the translation process that is directly related to the epigenetic activity of the body [26].

Aglaithioduline and SAHA are both histone deacetylase inhibitors (HDACi) regularly used in cancer treatments. Both of these drugs have proven to be effective in treating various diseases which involve HDACs (histone deacetylases), but there are some key differences between them. Aglaithioduline is an orally administered HDACi that has been shown to have a high level of anti-inflammatory activity and that can inhibit the growth of tumor cells in a variety of cancer types. Studies have demonstrated that aglaithioduline has a higher selectivity for HDAC enzymes and a lower toxicity profile than other HDACi drugs. Aglaithioduline has also been shown to be effective in combination with other drugs and to have synergistic effects when combined with chemotherapy [27]. SAHA, on the other hand, is an intravenously administered HDACi. It has been used to treat a variety of cancers, including leukaemia and RA [28]. In summary, both aglaithioduline and SAHA are effective HDACi drugs used in various pathological conditions, including cancer and RA. All the known activities of the compound and the herb clearly show that the compound aglaithioduline is a compound of interest and that herbs can also be studied further for the elucidation of synergetic activities of other compounds of potential use in the treatment of RA.

## 4. Materials and Methods

### 4.1. Similarity Searching

To begin the process of similarity searching, 2,231,213 compounds from the UNPD database were downloaded in sdf format with their corresponding metadata. The reference compound to be searched for similarity is appended at the beginning of the multi-compound sdf file and the merged sdf file is further imported into R [29]. Similarity searching was carried out based on the fingerprints of compounds that are stored in the database in matrix form [30]. The fingerprint set of compounds acts as a searchable database that consists of compounds’ fingerprints [31]. The fingerprints of the compounds are supported by the atom pair database [32]. Similarity searching is further carried out using the Tanimoto similarity search index [33]. The output is given in the form of index values in decreasing order: the higher the value, the more similar the compound is to the reference compound. Using the existing algorithms and library, an R-based Shiny application was developed, which was used to carry out similarity indexing.

### 4.2. Docking Studies

Molecular docking of the highly similar phytocompounds in comparison with the structure of target protein HDAC8 (PDB id: 1T69) [34] was carried out using Autodock vina [35,36] with help of POAP implementation [37]. The POAP tool is a powerful program designed to facilitate protein–ligand docking. This tool is used to optimize the binding parameters of protein–ligand complexes by using the methods of free energy calculation, electrostatics and molecular dynamics. The binding site of the target protein was assigned based on the interaction of the co-crystallized structure with the ligand SAHA and also, based on the literature, showing the active sites and binding sites on the target protein. In addition, the target protein was subjected to P2RANK [38] analysis, which provides detailed information on binding sites and their ranking. P2RANK binding site assignment is a computational method for predicting and ranking the potential binding sites of a target protein. The method is based on several physicochemical parameters that measure the strength of the interactions between a target protein and its ligands. These parameters include the electrostatic, hydrophobic, Van der Waals and steric interactions between the two molecules. To rank the binding sites, the P2RANK method calculates a score for each potential binding site, based on these parameters. The compounds selected from the similarity indexing were listed and the chemical structures in sdf format were converted to pdbqt format using POAP, with energy minimization conducted using the steepest descent method [39]. The energy-minimized structures were used for docking studies with an exhaustiveness of 100 [40]. The top complex based on the binding energy was further considered for MD studies based on the interactions with the active site of the co-crystallized structure of HDAC8. The preADMET properties of the test compound and the standard drug were predicted using the pkCSM server. The prediction of ADMET properties is very important in understanding the drug-likeness and toxicity profile [41].

### 4.3. Molecular Dynamics Studies

The simulation systems in the study considered were the apoprotein, the target in complex with the standard drug and the target in complex with the compound obtained from similarity indexing. Simulations were carried out on Gromacs version 2019.4 [42]. The system for the simulations was subjected to 50,000 steps of steepest descent energy minimization to nullify the steric overlap. Furthermore, all the systems were applied to a two-step equilibration phase, namely NVT (constant number of particles, volume and temperature) and NPT (constant number of particles, pressure and temperature). NVT equilibration was run for 500 picoseconds (ps) to stabilize the temperature of the system, and NPT was run for 500 ps to stabilize the pressure of the system to be subjected to dynamics, in order to relax the system and maintain restraint on the protein. The temperature coupling [42,43] method was applied for the NVT ensemble, along with the constant coupling of 1 ps with 303.15 K. For NPT, Nosé–Hoover pressure coupling [44,45] was applied with the constant coupling of 1ps with 303.15 K under conditions of position restraints (h-bonds) by the selection of random seed. The calculation of electrostatic forces for NVT and NPT were carried out using the particle mesh Ewald method [46]. All the systems were subjected to a complete 100 nanosecond (ns) simulation under no restraint conditions, with an integration time step of 0.002 ps and an xtc collection interval of 5000 steps for 100 ps.

The analysis of the Gromacs trajectory files was carried out using Gromacs utilities. The trajectory’s root-mean-square deviation (RMSD) was calculated using “gmx rmsd” and root-mean-square fluctuation (RMSF) analysis was carried out using “gmx rmsf”. The radius of gyration was calculated using “gmx gyrate” to determine whether the system reached convergence over the 100 ns simulation. The solvent-accessible surface area (SASA) was calculated using the “gmx sasa” command to determine the area accessible by water in the protein, in which the ligand can move around bound with the target protein. The hydrogen bond counts for the protein–ligand complex in both the target in complex with standard drug and the target in complex with the compound resulted from similarity indexing [47].

### 4.4. MM/PBSA and Residual Decomposition Energy

The molecular mechanics Poisson–Boltzmann surface area (MM/PBSA) is a computational method used to study the thermodynamic properties of protein–ligand complexes. This approach calculates the free energy of a protein–ligand system by breaking it down into separate contributions from various energy terms, such as molecular mechanics, Poisson–Boltzmann electrostatics and solvation-free energies. The MM/PBSA method has been widely used in drug discovery and design, as it provides insights into the molecular interactions between a ligand and a protein target. This information can be used to design and optimize drug candidates for further development. In the current study, the relative binding energy and its contribution to individual residues were calculated using the MM/PBSA method by utilizing the “*g_mmpbsa*” tool. The parameters from past research were taken into account while calculating the binding energy. Using 50 representative snapshots, the binding energy was determined throughout the steady trajectory observed between 50 and 100 ns [48,49]. The MM/PBSA result summary comprises Van der Waals energy, electrostatic energy, polar solvation energy and total binding energy. Based on the low binding energy of the system the stability of the system can be determined.

### 4.5. Principal Component Analysis

The molecule’s rotational and translational motion using the “least square fit” to the reference structure was examined using MD trajectories. The eigenvalue related to each eigenvector indicates the energy contribution of that part to the motion. The projection of the trajectory on a specific eigenvector illustrates the “time-dependent movements” that the components perform in a specific vibrational mode [50,51]. The projection’s time average reveals the contribution of atomic vibration components to this mode of coordinated motion. The eigenvectors and eigenvalues of the trajectory were generated using the Gromacs in-built utilities “g_covar” by calculating and diagonalizing the covariance matrix. The “g_anaeig” tool was also used to analyze and illustrate the eigenvectors.

## 5. Conclusions

The similarity indexing approach of identifying compounds having activity similar to that of the existing standard is a highly efficient and accurate method. In this study, the standard drug used in patients with rheumatoid arthritis, SAHA, was taken as a reference molecule against the database of natural compounds to find similarity indexes. The similarity indexing method resulted in the identification of aglaithioduline as a compound with ~70% similarity, and further in silico studies with HDAC8 clearly showed a high stability in the system in comparison with the standard drug. The system was also found to be very compact based on the radius of gyration around the axis. Hydrogen bond contact analysis also revealed the high binding affinity of aglaithioduline with HDAC8. Based on the results obtained, *Aglaia leptantha* and *Aglaia edulis*, in which aglaithioduline is present abundantly, can be taken further for in vivo and in vitro studies as anti–arthritic treatments specifically.

## Figures and Tables

**Table 1:** Molecular properties of the compounds SAHA and aglaithioduline.

Descriptor	SAHA	Aglaithioduline
Molecular Weight	264.325	306.431
LogP	2.4711	2.1184
Rotatable Bonds	8	9
Acceptors	3	3
Donors	3	2
2D Structure	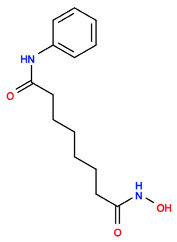	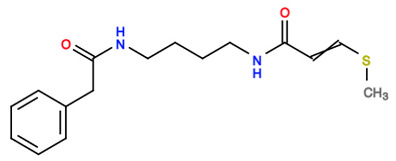

**Table 2:** The co-ordinates and the residues in the binding site of HDAC8 (1T69).

X	Y	Z	Residues
31.5106	−2.4886	−9.5104	ASP101 SER138 GLY139 GLY140 TRP141 HIS142 HIS143 GLY151 PHE152 CYS153 TYR154 HIS180 GLY206 PHE207 PHE208 MET274 GLY303 GLY304 TYR306 LEU31 LYS33 ILE34 ARG37

**Table 3:** Binding energy and amino acid interaction profile of HDAC target protein docked with aglaithioduline obtained from similarity indexing and the standard drug SAHA.

Compound Name	Docking Score (Kcal/mol)	Interaction Sites	
		Hydrophobic Interactions	Hydrogen Bonds
Aglaithioduline	−8.5	HIS180, PHE208	HIS143
SAHA	−8.7	TYR100, PHE152, PHE208	HIS180

## Data Availability

Data will be made available if required.

## Supplementary Materials

The following supporting information can be downloaded at: https://www.mdpi.com/article/10.3390/molecules28062430/s1, Figure S1: Protein structure of HDAC8 (PDB id: 1T69) with its structural and functionally important regions; Table S1: List of plants used for the treatment of rheumatoid arthritis across the globe; Table S2: The level of descriptors and the descriptors for each level; Table S3: ADMET properties of SAHA and aglaithioduline.
