# Supplementary material for: Exploring the Potential of Phytocompounds for Targeting Epigenetic Mechanisms in Rheumatoid Arthritis: An In Silico Study Using Similarity Indexing
Source: Molecules. 2023 Mar 7;28(6):2430. doi: 10.3390/molecules28062430 (PMC10051859; doi:10.3390/molecules28062430)
Supplement: Supplementary file 1 [file molecules-28-02430-s001.zip › molecules-2251939-supplementary.pdf]

Article

Exploring the Potential of Phytocompounds for Targeting Epigenetic Mechanisms in Rheumatoid Arthritis: An In Silico Study Using Similarity Indexing

Sanjay H. Deshpande [1], Zabin K. Bagewadi [1,*], T. M. Yunus Khan [2], Mater H. Mahnashi [3,*], Ibrahim Ahmed Shaikh [4], Sultan Alshehery [2], Aejaz A. Khan [5], Vishal S. Patil [6] and Subarna Roy [6]

[1] Department of Biotechnology, KLE Technological University, Hubballi 580031, India
[2] Department of Mechanical Engineering, College of Engineering, King Khalid University, Abha 61421, Saudi Arabia
[3] Department of Pharmaceutical Chemistry, College of Pharmacy, Najran University, Najran 66462, Saudi Arabia
[4] Department of Pharmacology, College of Pharmacy, Najran University, Najran 66462, Saudi Arabia
[5] Department of General Science, Ibn Sina National College for Medical Studies, Jeddah- 22421, Saudi Arabia
[6] ICMR—National Institute of Traditional Medicine, Belagavi 590010, India
* Correspondence: zabinb@gmail.com (Z.K.B.); matermaha@gmail.com (M.H.M.)

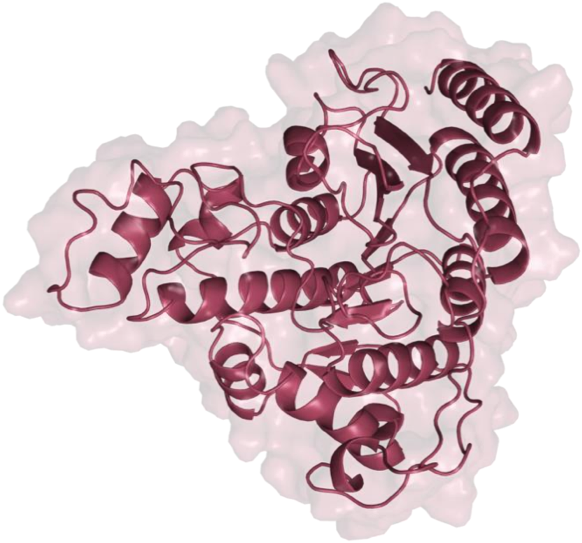

Figure S1. Protein structure of HDAC8 (PDB id: 1T69) with its structural and functionally important regions.

Table S1. List of plants used for the treatment of Rheumatoid arthritis across the globe.

Plant Names		
Abies Webbiana	***Calophyllum inophyllum***	*Garcinia mangostana*
Aconitum chasmanthum	*Capparis decidua*	*Glycyrrhiza glabra*
Aconitum deinorrhizum	*Capsicum frutescens*	*Glycyrrhiza uralensis*
Aconitum Falconeri	*Citrus aurantium*	*Hygrophila auriculata*
Aconitum laciniatum	*Coptis chinensis*	*Linum usitatissimum*
Actea Spicata	*Coptis japonica*	*Momordica charantia*
Aesculus indica	*Costus speciosus*	*Paeonia lactiflora*
Anisomeles malabarica	*Cucurbita aurantia*	*Platycodon grandiflorus*
Apium Graveolens	*Cucurbita pepo*	*Salix alba*
Balsamodendron Mukul	*Curcuma longa*	*Semecarpus anacardium*
Berberis vulgaris	*Dalbergia cancedaria*	*Tanacetum vulgare*
Boerhavia diffusa	*Eclipta alba*	*Withania somnifera*
Borago officinalis	*Equisetum arvense*	
Boswellia serrata	*Fraxinus hookeri*	

Table S2. The level of descriptors and the descriptors for each level.

Level	Descriptors
1D Properties	Molecular Weight, logP, PSA
2D Properties	Fingerprints, topological indices, maximum common substructures
3D Properties	Molecular fields, Shape

Table S3. ADMET properties of SAHA and Aglaithioduline.

Property	Model Name	SAHA	Aglaithioduline	Unit
Absorption	Water solubility	-2.217	-3.099	Numeric (log mol/L)
Absorption	Caco2 permeability	0.8	1.466	Numeric (log Papp in 10^{-6} cm/s)
Absorption	Intestinal absorption (human)	88.65	93.551	Numeric (% Absorbed)
Absorption	Skin Permeability	-3.052	-3.197	Numeric (log Kp)
Absorption	P-glycoprotein substrate	Yes	Yes	Categorical (Yes/No)
Absorption	P-glycoprotein I inhibitor	No	No	Categorical (Yes/No)
Absorption	P-glycoprotein II inhibitor	No	No	Categorical (Yes/No)
Distribution	VDss (human)	-0.144	-0.022	Numeric (log L/kg)
Distribution	Fraction unbound (human)	0.333	0.173	Numeric (Fu)
Distribution	BBB permeability	-0.806	-0.329	Numeric (log BB)
Distribution	CNS permeability	-2.811	-2.831	Numeric (log PS)
Metabolism	CYP2D6 substrate	No	No	Categorical (Yes/No)
Metabolism	CYP3A4 substrate	No	Yes	Categorical (Yes/No)
Metabolism	CYP1A2 inhibitior	No	No	Categorical (Yes/No)
Metabolism	CYP2C19 inhibitior	No	No	Categorical (Yes/No)
Metabolism	CYP2C9 inhibitior	No	No	Categorical (Yes/No)
Metabolism	CYP2D6 inhibitior	No	No	Categorical (Yes/No)
Metabolism	CYP3A4 inhibitior	No	No	Categorical (Yes/No)

Excretion	Total Clearance	0.408	0.132	Numeric (log ml/min/kg)
Excretion	Renal OCT2 substrate	No	No	Categorical (Yes/No)
Toxicity	AMES toxicity	No	No	Categorical (Yes/No)
Toxicity	Max. tolerated dose (human)	0.813	0.329	Numeric (log mg/kg/day)
Toxicity	hERG I inhibitor	No	No	Categorical (Yes/No)
Toxicity	hERG II inhibitor	No	No	Categorical (Yes/No)
Toxicity	Oral Rat Acute Toxicity (LD50)	2.113	2.353	Numeric (mol/kg)
Toxicity	Oral Rat Chronic Toxicity (LOAEL)	2.885	1.807	Numeric (log mg/kg_bw/day)
Toxicity	Hepatotoxicity	No	No	Categorical (Yes/No)
Toxicity	Skin Sensitisation	No	No	Categorical (Yes/No)
Toxicity	T.Pyriformis toxicity	0.54	1.447	Numeric (log ug/L)
Toxicity	Minnow toxicity	1.067	1.27	Numeric (log mM)
